# Transsexual Mastectomy: Selection of Appropriate Technique According to Breast Characteristics

**DOI:** 10.4274/balkanmedj.2016.0093

**Published:** 2017-03-28

**Authors:** Hüsamettin Top, Serkan Balta

**Affiliations:** 1 Department of Plastic, Reconstructive and Aesthetic Surgery, Trakya University School of Medicine, Edirne, Turkey

**Keywords:** Female-to-male, transsexual, subcutaneous mastectomy

## Abstract

**Background:**

Subcutaneous mastectomy for female- to-male transsexuals is usually the first surgical pro- cedure in sexual reassignment. The main objective of subcutaneous mastectomy is to create an aesthetically pleasing male chest contour by removing all glandular tissue while minimizing chest wall scars.

**Aims:**

In this paper, we present our experience with subcutaneous mastectomy performed in female-to- male transsexual patients. The authors recommend their point of view to aid in selecting the most suitable subcutaneous mastectomy technique depending on breast characteristics.

**Study Design:**

Retrospective cross-sectional study.

**Methods:**

Between March 2011 and December 2014, 52 patients underwent bilateral subcutaneous mastec- tomies (total of 104 mastectomies), performed using the following four techniques: Webster semicircular, concentric circular, vertical, and apron flap. The tech- nique decision depended on the breast size, degree of skin excess, skin elasticity, chest width, nipple areolar complex size and position.

**Results:**

Seventeen patients (32.7%) were operated with Webster semicircular, 7 patients (13.5%) with con- centric periareolar, 12 patients with vertical (23%); and 16 patients (30.8%) with the apron flap technique. The overall postoperative complication rate was 13.4%. All patients were satisfied with the aesthetic results of their subcutaneous mastectomies within the follow-up period.

**Conclusion:**

To obtain higher patient satisfaction with aesthetic results and lower postoperative complication rates, breast characteristics are evaluated in a detailed fashion, while choosing the ideal technique of Female-to-Male (FtM) subcutaneous mastectomy. The presented surgical new algorithm facilitates the selection of the most reliable surgical technique.

Female-to-Male (FtM) transsexualism is a gender identity disorder; these patients have a belief of having been created in the wrong body. These individuals suffer from persistent psychological discomfort related to their anatomical sex, and have a desire to live and be accepted permanently in the social role of male gender. The disturbance in transsexuals can be associated with physical conditions (e.g. intersex), sometimes a chromosome abnormality or any mental disorder, such as schizophrenia, and results in the impairment of social and occupational life activities. Due to their desire to change their anatomical sex characteristics to those of the opposite gender, sex reassignment surgery is supposed to be the main surgical treatment in these patients.

Bilateral subcutaneous mastectomy in FtM transsexuals is the one of the most important steps in gender reassignment operations, because achieving a male chest configuration with this first operation, importantly, facilitates living in the chosen gender role, particularly when they have large breasts. In these patients, mastectomy procedure involves the surgical removal of all gland tissue and the required amount of skin and subcutaneous fat tissue. Also reconstruction of the nipple-areolar complex (NAC) by proper positioning of the NAC and an adequate reduction of nipple and/or areola can be obtained, resulting in an esthetically pleasing male chest reconstruction. Although many studies have reported in the literature about mastectomy procedures regarding breast cancer and gynecomastia surgery, the techniques of subcutaneous mastectomy in FtM transsexuals have been mentioned in small number of papers, and six of them have been studied in large patient series (1,2,3,4,5,6). Generally, two main surgical options have been used in FtM chest contouring: breast reduction techniques can be modified or surgical methods developed for the treatment of gynecomastia performed with or without modification (2,3,4,5,6). Since the structure of the male breast differs from that of the female breast, not only in volume and projection but also in the size and position of NAC, subcutaneous mastectomy cases must be evaluated for appropriate surgical technique, preoperatively. The key determinants for choice of best technique are grade of skin excess and ptosis, breast size, and skin quality. To date, various mastectomy procedures in FtM transsexuals have been reported, including transareolar, semicircular, concentric circular, extended concentric circular, and nipple- areolar graft techniques (6,7,8,9,10,11). However, none of them has proposed the vertical pedicled subcutaneous mastectomy in FtM transsexuals. Also the apron flap technique of reduction mammoplasty has never been used as a term to define previous nipple-areola graft or free nipple graft techniques better. In this paper, we present our FtM transsexual cases who underwent subcutaneous mastectomy with four techniques.

## MATERIALS AND METHODS

The study was approved by the ethics committee of Trakya University. From March 2011 to December 2014, we performed 52 bilateral subcutaneous mastectomies at the Department of Plastic, Reconstructive and Aesthetic Surgery, Trakya University Hospital, Edirne, Turkey (Table 1). The average patient age was 28.2 (age range, 18-47). The weights of patients were between 40 and 100 kg and average body mass index (BMI) was 23.4 (BMI range, 15.6-36.7). Thirty patients had a history of receiving testosterone hormone therapy before operation. Hormonal therapy was discontinued 4 weeks before surgical intervention. Also, each patient underwent a complete psychiatric, endocrinological, urologic and gynecological evaluation, which allowed the diagnosis of “FtM transsexualism” to be made. An ultrasound examination was performed to evaluate the existence of glandular tissue and to exclude any pathologic mass in all cases. ‘Patient Informed Consent Form’ was taken from all individuals.

Preoperatively, the choice of technique was based on breast size and degree of skin excess, skin elasticity, NAC size and position. A four grade breast classification was performed according to breast size and skin excess:

A cup - minimal

B cup - moderate

C cup - large

D cup - significant

**Figure 1:**
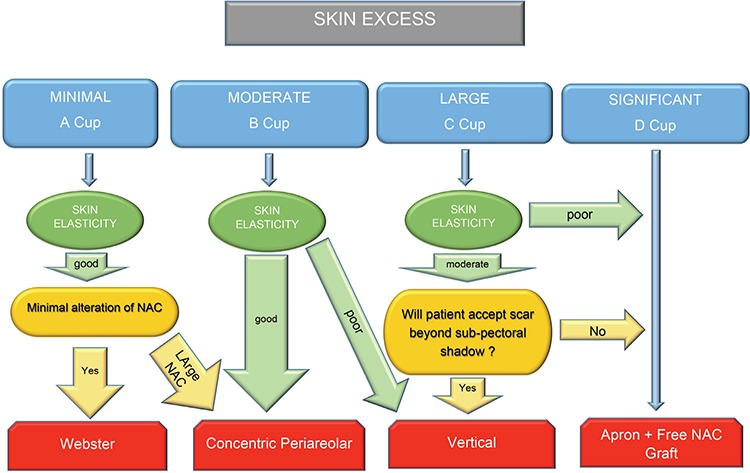
Diagram for selecting the most suitable subcutaneous mastectomy technique for female-to-male transsexuals. NAC: nipple areolar complex

All patients were informed about the appropriate technique regarding incisions and possible postoperative scars. For those patients who would not accept postoperative scars beyond the subpectoral shadow, this condition was noted. Then, specific subcutaneous mastectomy was chosen based on the algorithm presented in Figure 1. Appropriate antibiotics were administered 1 hour before the operation and for the 5 days after. A patient satisfaction questionnaire was performed 12 months after the surgery to evaluate the overall aesthetic result of subcutaneous mastectomy. Patient satisfaction was rated as “very good” (5), “good” (4), “satisfactory” (3), “less satisfactory” (2) and “unsatisfactory” (1).

### Operative procedure

All subcutaneous mastectomies were performed under general anesthesia. With the patient in prone position, specific skin incisions were made according to preoperative markings. During the elevation of flaps, extremely careful dissection was performed to preserve all subcutaneous fat above the mammary gland. Thus, adequate thickness skin produces an aesthetically pleasing chest wall appearance without strictly adhered and depressed areas between the skin and chest wall.

**Figure 2:**
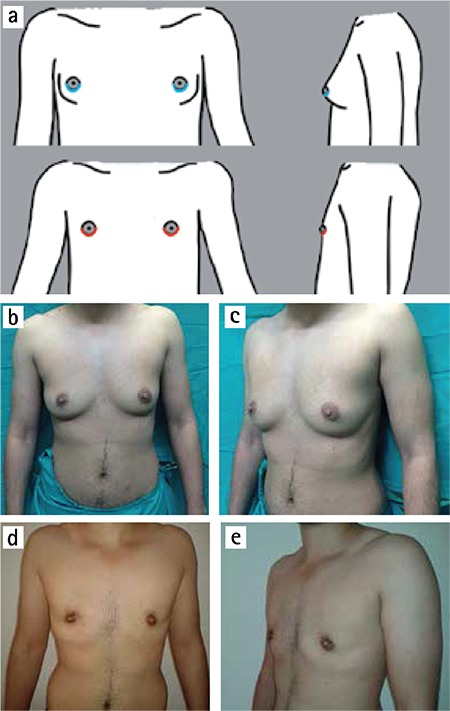
a-e. Webster semicircular technique. Incisions (above) and scars (below) (a). (b) and (c) preoperative; (d) and (e) postoperative views.

In the cases of breasts with minimal size and skin excess, the Webster technique was used (Figure 2). The incision starts from ‘three’ position in areola-skin conjunction and ends at ‘nine’ position. It can be extended laterally and medially by at least 1 cm, for better exposure of the subcutaneous dissection area. During elevation of NAC complex, a small amount of mammary tissue should be included under this flap to avoid depression deformity. Then, subcutaneous mastectomy is performed. At the end of this technique, in order to create an aesthetically pleasing masculine chest contour, the required amount of subcutaneous fat tissue can be excised from above the level of the submammary fold, if necessary. Although the technique is more challenging, because of the small exposure window through which dissection and hemostasis are performed, this small incision results in a well-concealed infra-areolar scar. Additionally, nipple reduction can be performed, if needed. A suction drain is placed in the subcutaneous dissection area. However, if the patient has the desire for a smaller areola, this technique can be revised to a concentric periareolar approach.

**Figure 3:**
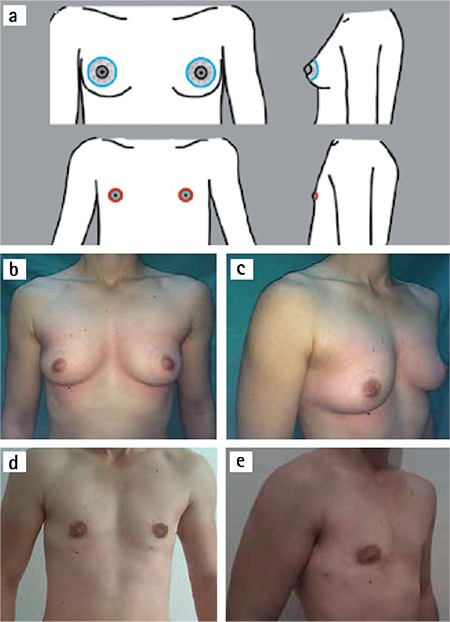
a-e. Concentric periareolar technique. Incisions (above) and scars (below) (a). (b) and (c) preoperative; (d) and (e) postoperative views.

The concentric periareolar technique can be used for breasts with moderate skin excess and good skin elasticity (B cup) or for breasts with minimal skin excess but which have a large NAC complex (Figure 3). Two concentric incisions are designed. The first is placed in the areola with the desired diameter to create a new NAC, and the bigger one is designed with a suitable diameter, enabling the excision of a sufficient amount of excess mammary skin. Then the area between two circles is deepithelialized to avoid compromising the blood supply to the NAC. Subcutaneous mastectomy is performed through a semicircular incision placed in the deepithelialized area. A sufficient amount of mammary tissue should be left beneath the NAC flap; this flap is carefully dissected with a wide-based dermal pedicle. If necessary, the submammary fold can be managed with resection of decided amount of subcutaneous fat tissue from above the level of this fold. A suction drain is placed in dissection cavity. The outer circle is then sutured to smaller one by the purse-string fashion. The technique gives good exposure window for dissection and hemostasis. Also it allows for the reduction of large areola and excess skin. Also, if nipple reduction is needed, it can be done in the same session because the vascular supply of this flap technique is mostly reliable.

**Figure 4:**
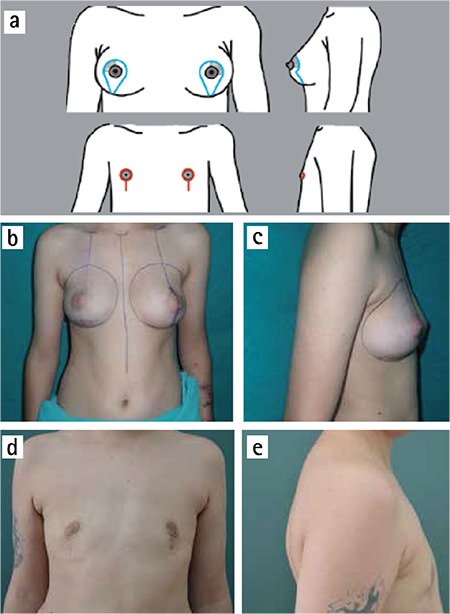
a-e. Vertical technique. Incisions (above) and scars (below) (a). (b) and (c) preoperative; (d) and (e) postoperative views.

We used vertical subcutaneous mastectomy for B cup breasts with poor skin elasticity and for breasts which have large skin excess (C cup) with moderate skin elasticity (Figure 4). The technique is similar to that described by Nahai (12). First, the ideal new position of the nipple is marked along the existing NAC line and between fifth and sixth ribs. Skin markings are made and deepithelialization is finished. Subcutaneous mastectomy is performed as only mammary gland excision with preserving all subcutaneous fat tissue. A suction drain is placed in the subcutaneous cavity. The advantages of this technique are that it facilitates the excision of breast tissue and hemostasis through a large window, and is also useful for a reduction of areola and skin envelope. The major drawback of this technique is that a vertical subareolar scar may extend beyond the subpectoral shadow. However, this scar can be well tolerated by the patients with chest hair.

**Figure 5:**
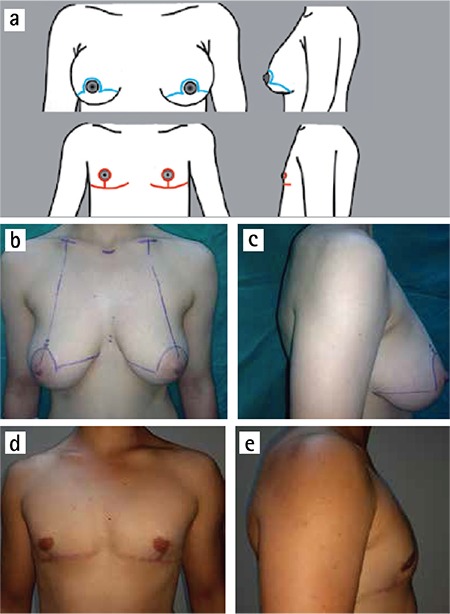
a-e. Apron flap technique. Incisions (above) and scars (below) (a). (b) and (c) preoperative; (d) and (e) postoperative views.

The apron flap technique has been proposed for breasts with large skin excess with poor skin elasticity (C cup) or for breasts with a significant skin excess (D cup) (Figure 5). Additionally, if the patient does not prefer the vertical scar that extends beyond the subpectoral shadow, this technique can be proposed. Preoperative markings are started with a lower incision. It is placed 1 to 2 cm above the inframammary fold. Then, upper incision is drawn on the skin part above the areola. In some cases, when the vertical length of apron flap is not enough, the center part of the upper incision can be extended like a semicircle towards the medial and lateral sides of the areola. Operation starts with harvesting the NAC full-thickness graft with a diameter of 2 to 3 cm followed by subcutaneous mastectomy. It is extremely important to preserve adequate thickness of subcutaneous fat during glandular tissue resection. The Apron flap is pulled down and temporarily sutured along the lower incision. The new position of NAC is marked along the existing NAC line, and the vertical height is placed approximately 2 cm above the inframammary suture line. However, the final position of the NAC graft can be adjusted after the patient sits up intraoperative to check the exact NAC position. A suction drain is placed under the apron flap. The disadvantages of apron flap technique are long subpectoral scar, nipple projection deformities and reduced sensation. The advantages are that it creates an excellent view for dissection and hemostasis, and also allows for a reduction of NAC and skin excess adequately.

## RESULTS

Fifty-two patients underwent bilateral subcutaneous mastectomies (total of 104 mastectomies) between March 2011 and December 2014. The techniques performed in these individuals included: Webster, 17 patients (32.7%); concentric periareolar, 7 patients (13.5%); vertical, 12 patients (2); apron flap, 16 patients (30.8%). Average resection weight was 349.5 g (range, 90-1310 g) for right breasts and 351.9 g (range, 80-1190 g) for left breasts. The pathological examination performed to all excised specimens and results is summarized in Table 1.

Complications were encountered in 7 patients (13.4%). These complications were divided in to 6 minor cases (11.5%), which could be managed nonoperatively; and 1 major case (1.9%), in which surgical intervention was necessary. Minor complications included minimal hematoma, 1 cases (1.9%); and superficial graft necrosis, 5 cases (9.6%). Small hematomas were aspirated and a pressure garment was applied for prevention. Superficial graft necrosis was treated with local wound care. Major complications included significant hematoma, in 1 case (1.9%); this hematoma was treated by reoperation and intense hemostasis. Follow-up ranged from 12 to 56 months (average, 28 months). The patient questionnaire revealed a high satisfaction level with the overall aesthetic outcome in all techniques (Table 2). Thirty-two patients (61.5%) rated the outcomes as “very good” (5), 13 patients (25%) said “good” (4), 5 patients (9.6%) reported “satisfactory” (3), and 2 patients (3.8%) were “less satisfactory” (2). No patients need a secondary revision procedure.

## DISCUSSION

Chest masculinization with subcutaneous mastectomy for female-to-male transsexuals is usually the first and most important step in the gender reassignment process after complete psychiatric assessment and endocrine treatment. The main goal of this procedure is to obtain an aesthetically pleasing chest contour with a minimal scar. Although the operative technique is thought to be similar to a gynecomastia correction or a mastectomy for breast disease in a female patient, subcutaneous mastectomy in FtM transsexuals is more difficult than both procedures because these individuals have considerably higher breast volume, a greater degree of skin excess and lower quality skin elasticity. Breast binding also negatively affects both skin excess and skin elasticity (6). Although various techniques have been published in the literature, describing how to perform an ideal chest contouring in FtM transsexual persons (1,2,3,4,5,6,7,8,9,10,11), there are only a few articles proposing an algorithmic surgical approach for subcutaneous mastectomy (4,6,10). In 1995, Hage et al. (4) published their algorithmic approach as the “Amsterdam experience” with three surgical options. Subcutaneous mastectomy by a transareolar technique combined with concentric periareolar deepithelialization was proposed in moderate skin excess cases. Cases with larger breast or severe ptosis were operated upon by modification of this method with skin excisions laterally and medially to NAC, sometimes by using a free NAC graft in combination with fusiform skin excisions. Monstrey et al. (6) shared their algorithm for chest wall contouring surgery in FtM transsexuals. They used five surgical techniques for mastectomy depending on skin envelope, breast size, degree of ptosis and skin elasticity as decisive factors: semicircular, transareolar, concentric circular, extended concentric circular and free NAC graft. In February 2015, Wolter et al. (10) proposed four surgical options for subcutaneous mastectomy. In cases with very small breasts and good skin elasticity without ptosis, they performed semicircular incision mastectomy in combination with liposuction. Grade I ptotic small breasts with moderate to poor skin elasticity were operated upon with a concentric circular approach. Cases with moderate to large breasts, ptosis grade II and moderate to poor skin elasticity underwent inferior pedicled mammoplasty. In cases with very large breasts, poor skin elasticity and grade III ptosis, a mastectomy with free NAC graft was performed. We performed subcutaneous mastectomy in 52 FtM transsexual patients (total of 104 mastectomies) in 4 years. Based on the results presented here and our experience, we propose an algorithm to help to decide upon the most suitable subcutaneous mastectomy technique in these individuals (Figure 1). In this algorithm, breasts are classified according to breast size, degree of skin excess and skin elasticity. NAC size and position are evaluated and postoperative scars are discussed with the patient. For small breasts (A cup) with minimal skin excess, good elasticity and normal NAC size, the Webster technique was performed. Webster first described this technique in 1946 for low-grade gynecomastia cases (13). As a FtM subcutaneous mastectomy technique, many authors have adopted this approach (3,4,5,6,7,8,9,10). The most important advantage of this technique is small and good concealed infraareolar scar. However, the small incision size makes the dissection between skin envelope and glandular tissue harder. Also, this restricted surgical access is more challenging in providing adequate hemostasis. Although NAC reduction can be combined with this technique, if NAC width is larger, the concentric periareolar technique should be selected. Due to good skin elasticity and minimal skin excess, the final result is very satisfactory for the patient and the surgeon, so secondary revisions are rarely required. In patients with moderate skin excess (B cup) or small breasts (A cup) that have large NAC, concentric periareolar technique was performed. This technique was first described by Davidson et al for gynecomastia cases in 1979 (14) and later adopted by Hage JJ in 1995 as a subcutaneous mastectomy procedure in FtM transsexuals (3,4). The advantages of this technique are possibility to reducing skin excess and large NAC, and to working through wide window for detailed dissection and delicate hemostasis. Skin elasticity should be carefully evaluated in breasts with moderate skin excess (B cup). If the skin elasticity is not good in these breasts, because of possibility of inadequately skin shrinkage, vertical subcutaneous mastectomy option should be used.

Cases with breast that has large skin excess (C cup) and that has moderate skin excess (B cup) with poor skin elasticity underwent subcutaneous mastectomy with vertical technique. The vertical scar mammoplasty technique was first described by Lotsch (15) in 1923 for mastopexy and was adopted later for breast reduction by Arie (16). In this study, we modified the technique described by Nahai (12) in 2005. This technique has never been proposed as a method for subcutaneous mastectomy in FtM transsexual chest contouring. It provides adequate surgical exposure during dissection between tissue planes and hemostasis. The new NAC complex position can be designed preoperatively. Neurovascular integrity and form of the NAC can be maintained. The desired amount of new infraareolar skin tissue can be excised both medial and lateral limbs of vertical closure with adjusting skin tightness of this area. However, in some cases with a thin subcutaneous flap covering the NAC neurovascular pedicle, contour deformity may remain below the NAC. Also, the lower end of the vertical scar may extend below the subpectoral shadow. The possibility of this final result should be discussed with patients and if they do not prefer this situation, an apron flap technique should be used.

For very large breasts (D cup) with significant skin excess and for breasts with a large skin excess (C cup) with poor elasticity, apron flap technique was adapted. Breast amputation with NAC graft technique has been proposed for very large breasts in FtM transsexuals (1,4,6,8,9,10). So far, apron flap technique has never been used to define this technique. Because an apron flap is created when making a dissection between subcutaneous fat and mammary gland tissue, we believe that this entitlement is more descriptive. This technique has many advantages: Subcutaneous mastectomy can be performed in a rapid fashion. The long incision allows good exposure for hemostasis and dissection between subcutaneous fat and glandular tissue. NAC can be adjusted desired size. Also, the lack of pedicle thickness below the NAC creates a more natural flattened male chest contour postoperatively.

Postoperative complications can include hematoma, superficial or full-thickness NAC graft necrosis, contour deformities, seroma, hypertrophic scar or keloid, and remaining skin excess. In our series of 52 bilateral subcutaneous mastectomies, our complication rate was 13.4%, which was similar to that reported in previous studies. Superficial graft necrosis was the most frequent complication (9.6%).

The patient questionnaire revealed a high satisfaction rate with the overall aesthetic outcome in all techniques (Table 2). Aesthetic outcome was rated as “very good” or “good” in 86.5% of cases.

In conclusion, our surgical approach facilitates the selection of most reliable technique for FtM subcutaneous mastectomy. Because the technique preference is highly dependent on breast size, degree of skin excess, skin elasticity, NAC size and position, all of these values were evaluated in a detailed and combined fashion. In this way, aesthetically pleasing male chest contour can be obtained with a lower postoperative complication rate.

## Figures and Tables

**Table 1:**
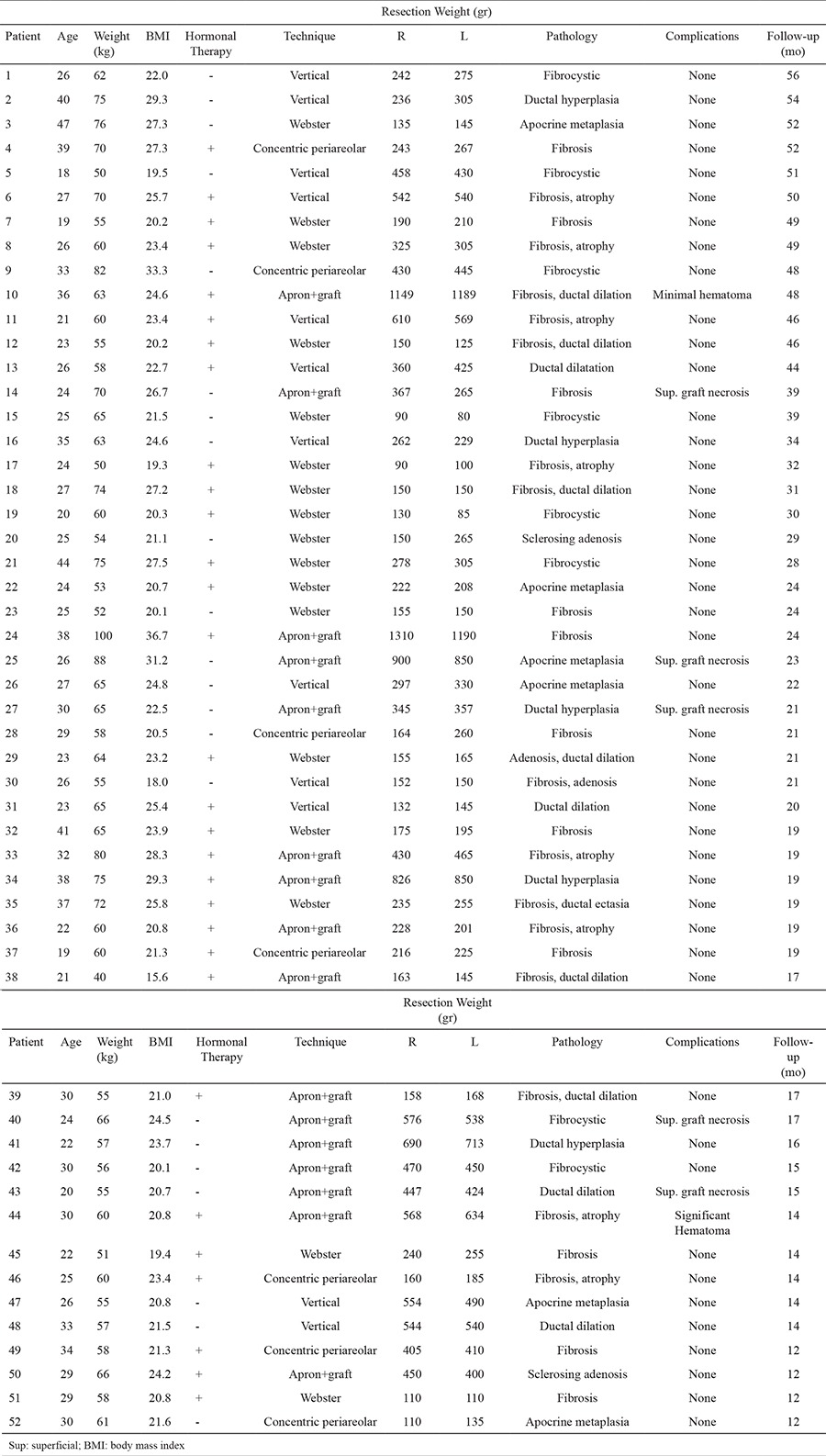
Patient characteristics

**Table 2:**
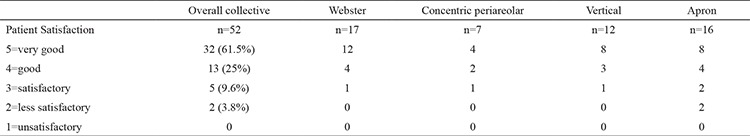
Overall aesthetic satisfaction
